# Synergy of Plant Essential Oils in Antibiotic Therapy to Combat *Klebsiella pneumoniae* Infections

**DOI:** 10.3390/ph16060839

**Published:** 2023-06-05

**Authors:** Mariana Romo-Castillo, Victor Andrés Flores-Bautista, Silvia Laura Guzmán-Gutiérrez, Ricardo Reyes-Chilpa, Mayra León-Santiago, Victor Manuel Luna-Pineda

**Affiliations:** 1 CONAHCYT/HIMFG, Laboratorio de Investigación en Inmunología y Proteómica, Hospital Infantil de México Federico Gómez, Mexico City 06720, Mexico; 2 Facultad de Estudios Superiores Zaragoza Campus II, Universidad Nacional Autónoma de México, Mexico City 09230, Mexico; victorflores31426@gmail.com; 3 CONAHCYT/Departamento de Inmunología, Instituto de Investigaciones Biomédicas, Universidad Nacional Autónoma de México, Mexico City 04510, Mexico; saguzmangu@conahcyt.mx; 4 Instituto de Química, Universidad Nacional Autónoma de México, Mexico City 04510, Mexico; chilpa@unam.mx (R.R.-C.); mayra.leon@iquimica.unam.mx (M.L.-S.); 5 Laboratorio de Investigación en Inmunología y Proteómica, Hospital Infantil de México Federico Gómez, Mexico City 06720, Mexico; luna.pineda@hotmail.com

**Keywords:** *Klebsiella pneumoniae*, essential oils, antibiotics synergic therapy, thyme, peppermint, turmeric, rosemary, plant-derived compounds, antibacterial infections

## Abstract

Increased antibiotic resistance presents a health problem worldwide. The World Health Organization published a list of pathogens considered a priority for designing new treatments. *Klebsiella pneumoniae* (Kp) is a top-priority microorganism, highlighting the strains that produce carbapenemases. Developing new efficient therapies or complementing existing treatments is a priority, and essential oils (EOs) provide an alternative. EOs could act as antibiotic adjuvants and enhance antibiotic activity. Employing standard methodologies, the antibacterial activity of the EOs and their synergic effect with antibiotics were detected. A string test was used to identify the impact of the EOs over the hypermucoviscosity phenotype presented by Kp strains, and Gas Chromatography–Mass Spectrometry analysis identified EOs and the composition of EOs. The potential of EOs for designing synergistic therapies with antibiotics to combat the infection of KPC diseases was demonstrated. In addition, the alteration of the hypermucoviscosity phenotype was shown as the principal mechanism of a synergic action between EOs and antibiotics. The differential composition of the EOs lets us identify some molecules that will be analyzed. Synergic activity of EOs and antibiotics can provide a solid platform for combating multiresistant pathogens that represent a severe health sector problem, such as Kp infections.

## 1. Introduction

Infection diseases are responsible for many illnesses and death worldwide, and last year, the World Health Organization reported high levels of resistant antimicrobial bacteria diseases in many countries. Principally, bloodstream infections caused by *Klebsiella pneumoniae* and *Acinetobacter* spp. increased above 50% (https://www.who.int/news/item/17-01-2020-lack-of-new-antibiotics-threatens-global-efforts-to-contain-drug-resistant-infections, accessed on 12 April 2023). *Klebsiella pneumoniae* (Kp) is a Gram-negative, encapsulated bacteria that possesses a lot of virulence factors (fimbriae, porins, siderophores, efflux pumps, type VI secretion system, biofilm, etc.) [1,2]. Kp is an opportunist pathogen that can cause infections at multiple sites such as the lung, urinary tract, bloodstream, brain, respiratory tract, and liver [3,4,5,6,7,8,9]. Its capsule acts as a protective shield that can avoid bactericidal action. In addition, the capsule is responsible for the viscosity and hypermucoviscosity phenotype of the strains cultured in nutrient media [10,11]. The genes involved in capsule synthesis are in a chromosomal pathogenesis island named the *cps locus* [12]. The capsule is involved in the resistance because it limits the interaction of antimicrobial molecules with the bacterial surface. The compounds that inhibit the capsule might help render capsulated bacteria [13].

Many Kp strains present an hypermucoviscosity phenotype, defined as when strains produce a thick filament of ~5 mm by the “string test”. This phenotype is commonly associated with infections such as bacteremia, liver abscesses, meningitis, and endophthalmitis [14]. The genes *magA* and *rmpA* have been associated with this phenotype [15,16]. It is important to remark that the hypervirulence of the strains is not always related to the hypermucoviscosity phenotype, but information about this correlation remains in continuous discussion. Some groups characterized hypervirulent strains (hvKP), identifying that only 60% have a hypermucoviscosity phenotype, but virulence between strains is comparable. This finding suggested that, contrary to the initial idea that hvKP strains possess an hypermucoviscosity phenotype hypervirulence is an additive characteristic of the strains, independent of their viscosity [17]. More studies are needed to understand the bacterial genetic factors responsible for the hypervirulent phenotype. Additionally, it is essential to note that the overexpression of many virulence factors, such as siderophores, capsules, lipopolysaccharides, and biofilm, causes the hyperviscosity phenotype.

The emergence of multidrug-resistant Kp strains has become a significant medical problem. Kp is intrinsically resistant to ampicillin because it possesses a penicillinase codified in its genome [18]. Kp develops antibiotic resistance faster than other strains because of its ability to acquire multiple mobile plasmids by horizontal transfer [19]. This genomic plasticity makes antibiotic therapies ineffective and indicates the need for developing alternative therapies against this pathogen. Many studies have demonstrated the prevalence and antibiotic resistance of Kp in strains. In Hangzhou, China, antibiotic resistance and the ratio of Kp cases increased by 30% in 2020 compared to 2006 [20]. A bloodstream infection analysis identified a significant increase in colistin Kp-resistant strains in recent years compared to the pre-SARS-CoV-2 period in many countries such as Brazil, China, India, Pakistan, Peru, South Korea, and Thailand [21]. Although antibiotic therapy is ineffective against SARS-CoV-2 infection, some studies stated that 70% of hospitalized patients received high-spectrum antibiotic therapy [22]. The main limitation in developing new antibiotics is financial efficiency. Since 2011, academics, organizations, and the pharmaceutical industry established that one of the potential strategies to combat antibiotic resistance is the development of antibiotic adjuvants.

Recently, natural-origin compounds, such as plant extracts and essential oils (EOs), have received particular attention because of their properties, economic viability, and efficacy. EOs are volatile molecules with antibacterial, anti-inflammatory, and antioxidant properties that effectively combat many diseases. EOs are synthesized by all plant organs (buds, flowers, leaves, stems, seeds, fruits, roots, etc.). Generally, EOs are liquid at room temperature, but some can be solid or have a resinous texture. The principal antibacterial activity of the EOs is caused by their chemical composition, principally by the presence of terpenoids. The global composition of EOs can vary in response to seasonal variation, culture conditions, the clime, and even the oil extraction method [23]. This variability is essential to consider because it can affect their antibacterial activity and the standardization of their application. However, EOs could be the best option to combat biofilm- and capsulated-related bacterial infections when compared with other plant-origin compounds. EOs can disrupt the bacterial capsule by their hydrophobicity, interact with the lipids, and disrupt the barrier making bacterial cells more accessible and permeable [24].

The effective antimicrobial efficacy of EO combinations has already been demonstrated [25]. However, the synergy between antimicrobial agents, plant extracts, and EOs is a new concept that has yet to be studied. A positive effect has been observed in using tetracycline and erythromycin with ethanolic mango peel extract (*Mangifera indica*) against *S. aureus* [26,27,28]. The methanolic extract of Damascus rose (*Rosa damascena*) decreases the minimum inhibitory concentration (MIC) of tetracycline against *P. aeruginosa* [29]. The effect of reducing the antibiotic MIC and their effective activity against some bacteria supports the idea that EOs could be used as an effective adjuvant to combat many bacterial diseases. This study aimed to demonstrate the potential of EOs to combat *Klebsiella pneumoniae* diseases as synergic therapy for antibiotics. This therapy could be successful for all Kp infections regardless of their antibiotic resistance or virulence level.

## 2. Results

### 2.1. Chemical Composition of EOs

Thirty-five plant EOs were screened to identify the best oil against Kp (data not shown), and only four EOs were included in this study (Table 1). A local producer from Coyoacan, Mexico City, Mexico, provided all EOs.

General major compounds of the essential oils were identified by comparison with available authentic samples. Still, their Kováts retention index was also obtained and compared with the reported data, supporting our chemical characterization of the tested oils. The major constituents of the EOs have been compiled in Table 2.

The major constituents of thyme EO were thymol (37.31%), o-Cymene (22.12%), and **γ**-Terpinene (11.97%) with a retention time (rt) between 8.4 to 13.3 and a match of 98.4 to 99.5; the minor constituent was p-Mentha-2,4(8)-diene (0.25%) with an rt of 9.63 and a match of 88.2. For rosemary EO, major constituents were eucalyptol (43.28%), (+)-2-Bornanone (13.34%), and **α**-pinene (13.17%), with an rt between 6.61 to 10.74 and a match of 99.3 to 99.8; while the minor constituent was isoterpinolene (0.31%) with an rt of 9.64 and a match of 91.7. For peppermint, the EO was composed majorly of menthol (38.19%), D-menthone (22.96%), and p-Mmenthan-1-ol (8.39%) with a retention time (rt) between 10.91 and 11.20 and a match of 95.2 to 99.8; the minor constituent was cis-Muurola-4(14), 5-diene (0.25%) with an rt of 16.45 and a match of 83.5. Finally, major constituents of turmeric EO were ar-turmerone (40.40%), turmerone (16.36%), and α-Curcumene (4.53%) with a retention time (rt) between 16.50 and 19.28 and a match of 97.7 to 99.0; the minor constituent was (+)-4-Carene (0.21%) with an rt of 9.64 and a match of 88.6.

### 2.2. Antimicrobial Activity of EOs against Kp Strains

**Figure 1:**
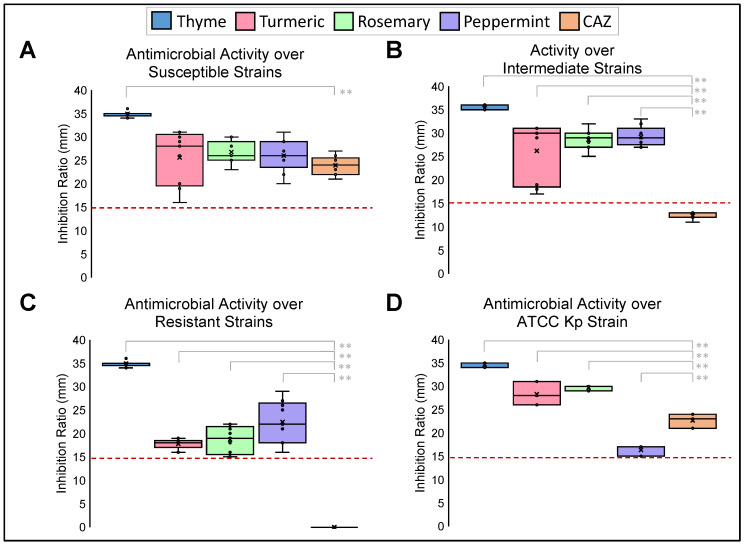
Antimicrobial Activity of EOs against different resistant profile Kp strains. Disc diffusion test of inhibition growth of Susceptible (**A**), Intermediate (**B**), Resistant (**C**), and reference ATCC 700603 (**D**) strains in the presence of EOs. Ceftazidime (CAZ) was used as a positive control. ** *p* < 0.01. Pointed line: Breakpoint is considered an effective antimicrobial activity for EOs. Median was identified by the line in each bar, points (⋅) represent each single data point and cross (x) represent outliers.

The antimicrobial activity of the EOs was tested against Kp clinical strains. These clinical strains were grouped following their antibiotic resistance profile in susceptible (Low Resistant Strain, LRS), intermediate (Medium Resistant Strain, MRS), and resistant (High Resistant Strain, HRS) groups. The antimicrobial activity test was performed by disc diffusion test in Mueller–Hinton agar. Inhibition ratios were measured for the four EOs, Ceftazidime, and dimethyl sulfoxide (DMSO). DMSO was used as an organic solvent to prepare dilutions and facilitate the solubilization of the EOs. The antimicrobial effect of the EOs was considered efficient when the tests showed a ratio ≥ of 15 mm, which was the breakpoint reported for Ceftazidime by the Clinical Laboratory Standards Institute (CLSI; https://clsi.org, accessed on 8 December 2022). A great antimicrobial activity by EOs was identified against all strains regardless of their antimicrobial resistance profile (Figure 1). Thyme EO has the best antimicrobial activity compared to other EOs and Kp clinical strains (*p* < 0.01) (Figure 1A). Turmeric, rosemary, and peppermint EOs have the same inhibition ratios as well as susceptible Kp strains; nevertheless, a significant statistical difference (*p* < 0.01) was identified when they were analyzed with intermediate and resistant Kp strains (Figure 1B,C).

**Figure 2:**
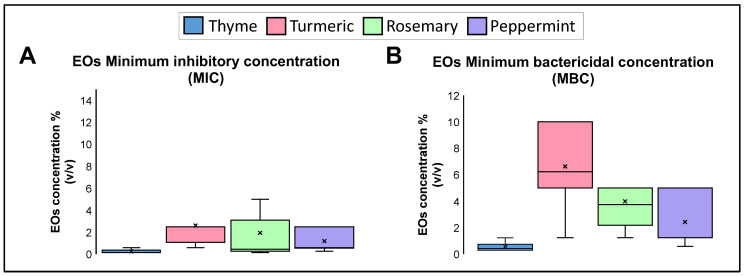
(**A**). Minimum inhibitory concentration (MIC) and (**B**). Minimum bactericidal concentration (MBC) of the EOs against Kp strains. Median was identified by the line in each bar, points (⋅) represent each single data point and cross (x) represent outliers.

A serial dilution test determined each EO’s minimum inhibitory concentration (MIC) and minimum bactericidal concentration (MBC). The MIC and the MBC vary in each group, regardless of its level of antibiotic resistance. Thyme is the best EO against Kp strains, with an MIC median of 0.15% (*v*/*v*) and an MBC median of 0.45%. Peppermint is another EO with bacteriostatic and bactericidal activity, with an MIC median of 0.60% and an MBC median of 1.25%. Rosemary EO had an MIC median of 0.45% and an MBC median of 3.75%, while turmeric had an MIC median of 2.50% and an MBC median of 6.25% (Figure 2). Non-statistical differences were shown between MIC and MBC groups.

### 2.3. Synergic Effect of EOs with Ceftazidime (CAZ)

**Figure 3:**
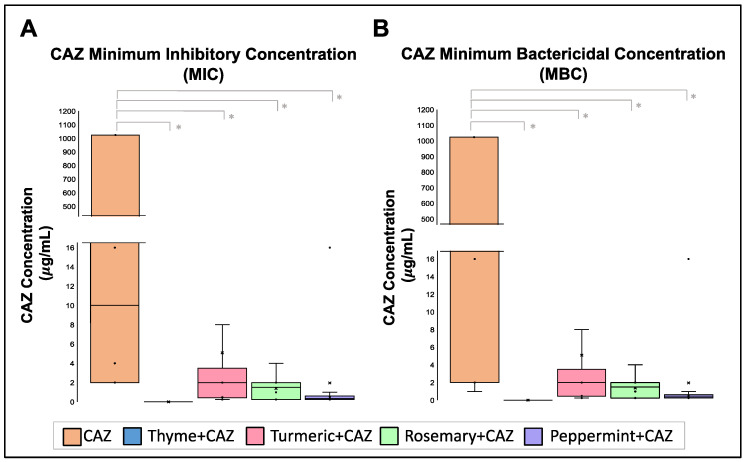
Effect of EOs on Ceftazidime (CAZ) MIC and MBC concentration. (**A**). Minimum Inhibitory Concentrations (MIC) and (**B**). Minimum Bactericidal Concentration (MBC) of Ceftazidime is reduced in the presence of the EOs. Median was identified by the line in each bar, points (⋅) represent each single data point and cross (x) represent outliers. * *p* < 0.05.

Antibiotics are the primary therapy used to control Kp infections; however, they are losing effectiveness. The hypermucoviscosity of Kp strains affects antibiotic diffusion and activity, affecting the control of these diseases. A checkerboard assay was performed to analyze the synergic effect that EOs could have with antibiotic therapy. The first antibiotic used was Ceftazidime (CAZ), recommended to control urinary diseases and nosocomial-associated pneumonia. The MIC median of CAZ was 32 μg/mL, and its MBC median was 10 μg/mL, but if an EO is combined with an antibiotic, the MIC and MBC values decrease significatively (*p* < 0.5) (Figure 3). Interestingly, the combination of CAZ and thyme was lethal to all the strains, still in very low concentrations of 0.12 μg/mL: 0.1% (CAZ:thyme). Peppermint EO at 0.22% reduced the CAZ MIC median to 0.5 μg/mL and MBC median to 0.38 μg/mL. Rosemary at 0.22% reduced the CAZ MIC median to 0.75 μg/mL and MBC median to 1.50 μg/mL. In comparison, turmeric at 0.63% decreased CAZ MIC median to 0.50 μg/mL and MBC median to 2.00 μg/mL (Figure 3).

When the Fractional Inhibitory Concentration Index (FICI) was calculated for each combination, a synergic effect was demonstrated between CAZ + thyme, with a FICI mean of 0.250, and in the CAZ + peppermint mixture, with a FICI mean of 0.357. At the same time, turmeric and rosemary EOs present only an additive effect with FICI mean between 0.536 to 0.545 (Table 3).

### 2.4. Synergic Effect of EOs with Gentamicin (GEN)

**Figure 4:**
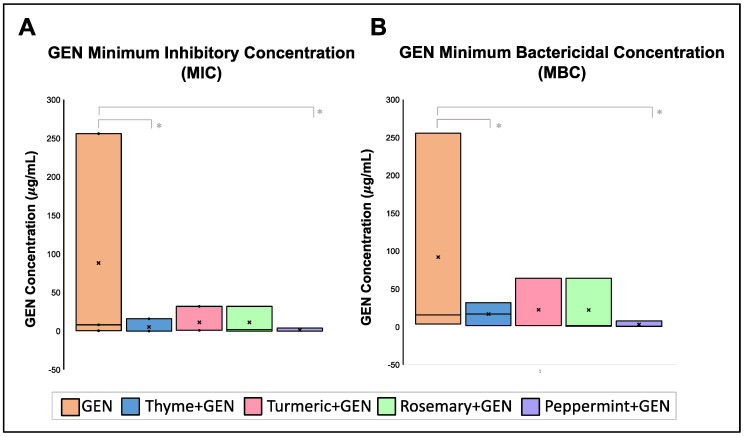
Effect of EOs on Gentamicin (GEN) MIC and MBC concentration. (**A**). Minimum Inhibitory Concentrations (MIC) and (**B**) Minimum Bactericidal Concentration (MBC) of GEN is reduced in the presence of the EOs. Median was identified by the line in each bar, points (⋅) represent each single data point and cross (x) represent outliers. * *p* < 0.05.

To identify the potential of EOs as synergic therapy with antibiotics, a second EO antibiotic was probed. The second antibiotic analyzed was Gentamicin (GEN), used for severe blood, bone, gastrointestinal, urinary, and meningitis infections. For this experiment, strain 889U (sensible to GEN), strain 98LCR (intermediately resistant to GEN), and strain 197U (resistant to GEN) were employed. The principal limitation to analyzing more strains is that our collection lacks other intermediate strains. The MIC median of GEN was 8 μg/mL, and it had an MBC median of 16 μg/mL, but EOs combined with an antibiotic decreased the MIC and MBC values of GEN (*p* < 0.5) (Figure 4). The combination of GEN and thyme was not lethal, but in 0.08% of Thyme EO, the MIC and MBC of GEN decreased to 16 μg/mL. Peppermint EO at 0.30% reduced the GEN MIC median to 1.0 μg/mL and its MBC median to 2.0 μg/mL. Rosemary at 0.15% reduced GEN MIC and MBC medians to 2.0 μg/mL. Finally, turmeric at 1.25% decreased GEN MIC median to 1.0 μg/mL and MBC median to 2.00 μg/mL.

When the Fractional Inhibitory Concentration Index (FICI) was calculated for each EO combination with GEN, a synergic effect was demonstrated between GEN + peppermint with a FICI mean of 0.275, GEN + thyme with a FICI mean of 0.410, and GEN + rosemary mixture with a FICI mean of 0.458. Turmeric presented an indifferent effect with GEN with a FICI mean of 1.083 (Table 4).

### 2.5. Synergic Effect of EOs with Ciprofloxacin (CIP)

**Figure 5:**
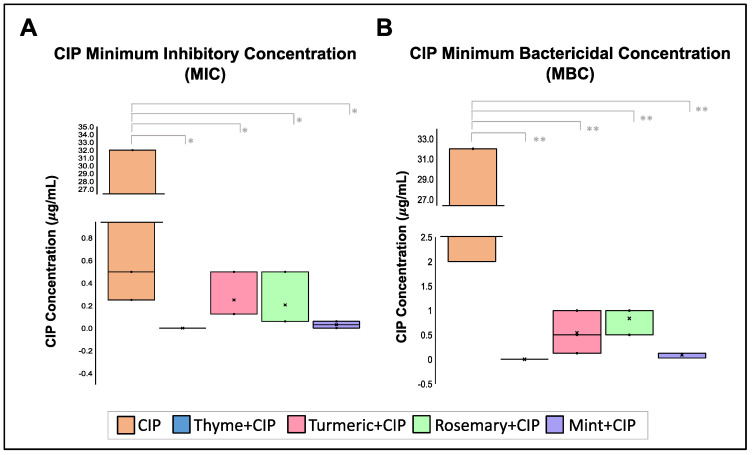
Effect of EOs over Ciprofloxacin (CIP) MIC and MBC concentration. (**A**). Minimum Inhibitory Concentrations (MIC) and (**B**). Minimum Bactericidal Concentration (MBC) of GEN is reduced in the presence of the EOs. Median was identified by the line in each bar, points (⋅) represent each single data point and cross (x) represent outliers. * *p* < 0.05, ** *p* < 0.01.

Finally, a third antibiotic was probed. Ciprofloxacin (CIP) treats urinary tract infections, pneumoniae, and skin and bone infections. For this experiment, two new strains were employed (817LCR and 910LCR) because all of the strains included those who are sensible to CIP. Although 817LCR and 910LCR strains have no complete antibiotic profile, intermediate and resistant profiles to CIP were detected, respectively. As for previously analyzed antibiotics, results demonstrated that EOs decrease CIP MIC and MBC (Figure 5). The MIC median of CIP was 0.5 μg/mL, and its MBC median was 2 μg/mL. Interestingly, as found for CAZ, the combination of CIP and thyme was lethal to all the strains, still in very low concentrations of 0.15 μg/mL:0.08% (CIP:thyme). Peppermint EO at 0.30% reduced the CIP MIC median to 0.125 μg/mL and the MBC median to 0.30 μg/mL. Rosemary at 0.15% reduced the CIP MIC median to 0.10 μg/mL and MBC median to 1.00 μg/mL. In comparison, turmeric at 0.1.25% decreased the CIP MIC median to 0.06 μg/mL and the MBC median to 0.13 μg/mL (Figure 5).

When the Fractional Inhibitory Concentration Index (FICI) was calculated for each EO combination with CIP, a synergic effect was demonstrated between CIP + peppermint with a FICI mean of 0.3.11, CIP + thyme with a FICI mean of 0.389, and CIP + rosemary mixture with a FICI mean of 0.459. Turmeric presents an additive effect with CIP with a FICI mean of 0.835 (Table 5).

### 2.6. Effect of EOs over the Hypermucoviscosity Phenotype of Kp Strains

**Figure 6:**
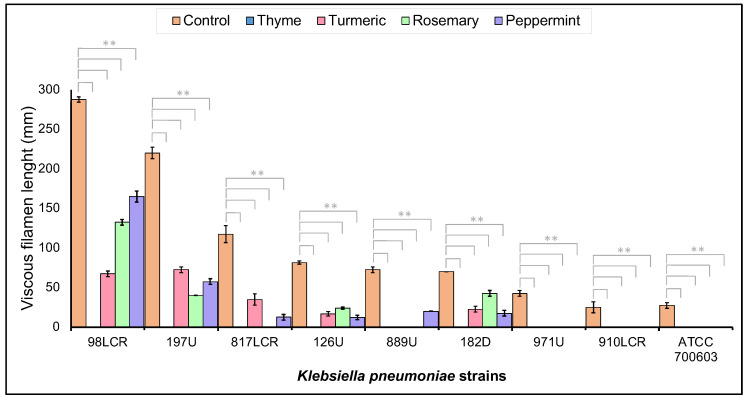
Decrease in hypermucoviscosity phenotype in Kp strains induced by EOs. String test of hypermucoviscosity strains in the absence (Control) and presence of EOs. ** *p* < 0.01.

The emergence of Kp hypermucoviscosity strains is associated with the increasing antibiotic resistance phenotype. The hypermucoviscosity phenotype enhances resistance to antibiotics by minimizing the binding of antibiotics to the bacterial surface [30]. To understand EOs’ action mechanism, their effect on the hypermucoviscosity phenotype was evaluated by a string test. Results demonstrate that EOs can decrease the hypermucoviscosity phenotype in all the strains, and some EOs can even eliminate it, such as thyme (Figure 6). Turmeric EO reduces the hypermucoviscosity with a mean of 84.56 ± 14.98% (in a range of 67.05 to 100%), rosemary EO decreases the hypermucoviscosity with a mean of 82.84 ± 23.98% (in a range of 39.29 to 100%), and peppermint EO reduce the hypermucoviscosity with a mean of 82.06 ± 18.65% (in a range of 42.61 to 100%). This effect on the hypermucoviscosity phenotype is independent of the bacterial resistance pattern. This result could suggest that the physical barrier presented by this hypermucoviscosity phenotype could make bacteria more vulnerable to environmental stimuli such as the presence of antimicrobial agents.

## 3. Discussion

Antibiotic resistance is a global problem that affects the health sector. The principal origin of antibiotic resistance appears to be from extended hospital stays and indiscriminate antibiotic use. The SARS-CoV-2 pandemic could influence the emergence of multiresistant pathogens because of the uncontrolled application of antibiotics. Kp is a common opportunist pathogen causing many hospital-patient-associated infections, and its antibiotic resistance is increasing faster than expected. The treatment of Kp-associated diseases has become problematic because of the emergence of multidrug-resistant strains. For this reason, it is included at the top of the priority list of the WHO to develop new treatments against, and plant-origin compounds are gaining attention.

Plant-origin compounds provide many opportunities to identify and design new effective therapies against multiresistant bacterial infections. EOs gained interest as an effective alternative against multiresistant diseases. Initial screening with 35 EOs was performed, but only 4 were included owing to their inhibition ratio >15mm, which were thyme, turmeric, rosemary, and peppermint EOs. These EOs’ bactericidal activity is comparable with antibiotics and acts against HRS, and their composition is similar to other EOs. Although EOs are a product of the secondary metabolism of plants, which are produced to make plants competitive in their environment, many factors could affect their chemical composition. These factors are classified into exogenous and endogenous factors. The exogenous factors are the environmentally regulated factors, such as light, precipitation, growing site, and soil, which might modify the amount of the volatile compounds of the EOs. The endogenous factors are strictly related to the anatomical and physiological characteristics of the plants linked to the chemical variation metabolism of the different parts of the plant, the development stage, and the genetically related factors [23,31]. Other factors that can influence the composition of an EO are the extraction method, the selection of the solvent used for the extraction, the application of pre-distillation treatments, and the temperature used in the extraction, as well as the mechanical and physical conditions used for the obtaining and conservation of the plant samples before the extraction of the EO [32,33]. Thymol is thyme’s major component; in other studies, it has shown inhibitory effects against Gram-negative and Gram-positive bacteria strains [33,34]. Thyme is the best option for designing a synergic therapy against Kp infections considering the results obtained in this study. Eucalyptol was identified as a significant component of rosemary EO and has been demonstrated to inhibit the growth of bacteria such as *S. aureus*, *P. aeruginosa*, *E. coli*, *E. faecalis*, and Kp ATCC 700603, as well as the yeast *C. albicans* [35]. Menthol is another component with antimicrobial activity against pathogenic and non-pathogenic microorganisms, such as *S. aureus*, *S. mutans*, *E. faecalis*, *S. pyogenis*, *L. acidophilus*, *P. aeruginosa,* and the yeast *C. albicans* [36]. It was the major component of Peppermint EO. Ar-turmerone is the major component of turmeric EO and has anti-inflammatory, anti-plasmodial, neuroprotective, and anti-aging activities. Nevertheless, there are indications of its antioxidant and antibacterial activity. The composition of thyme EO was comparable with the composition previously reported, although the amount of some components in other EOs differs [37]. For rosemary EO, the amount of Linalool (13.34%) was higher for other reports that present 0.4–0.5% [38,39]. Peppermint EO presents more p-Menthan-1-ol (8.39%) than other reports, with only 0.3–0.9% [40]. Curcumin has been reported as the main component of turmeric; however, ar-turmerone was identified in this study [41]. Recognizing the potential of plant-origin compounds can lead us to develop new strategies quickly that help us combat multiresistant diseases. Despite the differences identified in the concentration of some compounds, it is essential to determine the presence of these compounds to identify the molecules responsible for the antimicrobial activity that has been reported. This information will help identify specific EO molecules that can be used to combat different pathogens and, in combination with other compounds, in the appropriate concentrations, favor the design of synergistic therapies and allow their implementation at an industrial level. Metabolic engineering and nanotechnology have significantly advanced the synthesizing of some specific compounds, created release systems, and even obtaining synthetic EOs with natural EOs’ benefits.

The top result in this study was the synergic effect that EOs have with antibiotics of different families, such as aminoglycosides, quinolones, and cephalosporins. However, the synergic effect of the EOs is different. The best synergic effect between EOs and antibiotics was confirmed for thyme with CAZ and peppermint for GEN and CIP. CAZ is a third-generation cephalosporin that inhibits enzymes responsible for cell wall synthesis. Gentamicin is an aminoglycoside that produces membrane disruption and impaired protein synthesis. CIP is a second-generation fluoroquinolone that blocks DNA gyrase and Topoisomerase IV activities, preventing DNA replication [42]. A study using eucalyptol, amoxicillin/clavulanic acid (AMC), and Gentamicin showed a synergistic effect against *S. aureus* from osteomyelitis patients when compared with the combination of AMC with Gentamicin, which did not produce an impact [43]. The study supports the claim of the initial analysis using alternative plant compounds, such as EOs, to design alternative and complementary therapies to combat Kp diseases. The results suggest that a mixture of low concentrations of antibiotics and EO combinations could fight Kp infections, even if multiresistant or hypermucoviscosity strains cause them. It supports the idea that EOs affect the physical barrier responsible for the hypermucoviscosity phenotype allowing the internalization of antibiotics and acting against the pathogen. The complex composition of EOs in bioactive components and their liposoluble nature helps penetrate the biofilm, capsule, and bacterial membranes, altering multiple pathways that help control many diseases [44]. A comparison of the antimicrobial activity of EOs with sensitive, intermediate, and resistant Kp strains shows similar levels of activity, suggesting that the mechanisms used by bacteria to generate resistance to antibiotics are inefficient in providing resistance to EOs. These approaches also could help design therapies, nanoparticles, and textiles useful in biomedicine to control and combat other bacterial infections and reduce the use of antibiotics [45,46,47]. The therapeutic efficacy of Gentamicin and Ceftazidime co-encapsulated into liposomes showed an additive effect on rat survival, followed by a single dose or as a 5-day treatment [48].

This study has some limitations that should be considered. Although the direct effect of EOs against Kp is tested during this study, it is necessary to analyze if the volatile compounds present during evaporative diffuser use have the same effect. Commonly, EO application is performed through these kinds of diffusers, and the dosage and composition of airborne components will change and modify EO antimicrobial activity, so future studies are needed to analyze the employment of EO synergic treatment through aerial administration to combat respiratory infections caused by Kp. However, this study provides strong evidence of synergic EO therapy’s effectiveness.

## 4. Materials and Methods

### 4.1. Essential Oils

The essential oils were obtained from a local Coyoacan, México City, producer. The plants were cultivated in organic conditions (without synthetic fertilizers and pesticides), and essential oils were obtained by steam distillation following published methodology [49] from their leaves, stems, flowers, and rhizomes, as described in Table 1. The oils were collected, deposited in amber vials, and stored at 4 °C.

### 4.2. Chemical Analysis

The essential oil in chloroform solution was analyzed by gas chromatography (GC) using an Agilent 7890N coupled to an Agilent Technologies 5977A mass spectrometer GC(MS). The injection volume was 1 µL, split 20:1. The capillary column was an HP5-ms 30 m × 250 µm ID × 0.25 µm film thickness; the carrier gas was helium, flow rate, 1 mL/min; oven temperature range was from to 40 to 300 °C (Program: 40 °C, 1 min ramp 1, 8 °C/min; 200 °C, 1 min ramp 2, 15 °C/min; 300 °C, 2 min) along 4 min; injector temperature was 280 °C; and detector temperature was 310 °C. Mass spectra were registered over *m*/*z* 30–600 using an ionizing voltage of 70 eV. The essential oil constituents were characterized by matching their mass spectra with the compound library NIST (National Institute of Standards and Technology, U.S. Department of Commerce, Gaithersburg, MD, USA). The alkanes C8 to C24 were used to calibrate the Kováts scale (lineal retention indices), and experimental results were compared with reported data (NIST). The monoterpenes α-pinene, β-pinene, 3-carene, D-limonene, eucalyptol, γ-terpinene, linalool, α-terpineol, citronellol, nerol, L-carvone, linalyl acetate, thymol, carvacrol, neryl acetate, and geranyl acetate were also identified by comparison with mass spectra of authentic samples.

### 4.3. Microorganisms

*Klebsiella pneumoniae* clinical strains were used to analyze the potential of EOs. Previously characterized strains [50] were classified and selected based on their resistance pattern (Table 6). Low-resistance strains (LRSs) were defined as strains that are sensitive to Ceftazidime and only are resistant to one antibiotic. Medium-resistance strains (MRSs) are intermediately resistant to Ceftazidime (CAZ) and five other antibiotics. Last, high-resistance strains (HRSs) were defined as resistant to CAZ and seven other antibiotics. The antibiotics analyzed were cefpodoxime (10 μg), ceftazidime (30 μg), cefepime (30 μg), ceftriaxone (30 μg), aztreonam (30 μg), Gentamicin (10 μg), amikacin (30 μg), ciprofloxacin (5 μg), ofloxacin (10 μg), meropenem (10 μg), imipenem (10 μg), and trimethoprim/sulfamethoxazole (1.25/23.75 μg) by the Kirby–Bauer method. Resistance patterns were determined according to the Clinical Laboratory Standards Institute (https://clsi.org, accessed on 8 December 2022). The *K. pneumoniae* ATCC 700603 strain was used as a control. Three LRSs (640U, 889U, and 126U), three MRSs (98LCR, 338U, 160D), and three HRSs (971U-1, 197U, and 182D) were included in the first part of the study. Then, two strains were added for Ciprofloxacin synergy analysis because of their intermediate (817LCR strain) and resistant (910LCR strain) pattern against CIP, although they possessed an incomplete antibiogram analysis. Strains were grown in Brain Heart Infusion agar (BHI) at 37 °C for 18 h.

### 4.4. Antimicrobial Activity Test

Antibacterial activity against Kp strains was evaluated by the disk diffusion method in Mueller–Hinton Agar (MHA). Briefly, each strain was cultured in 5 mL of Mueller–Hinton broth (MHB) for 18 h at 37 °C and 150 rpm. Then, overnight cultures were diluted at a turbidity of 0.5 McFarland (108 CFU/mL). Then, 100 μL of the bacterial inoculum was uniformly spread in MHA, and blank-standard disks were impregned with 10 μL of each EO. Plates were incubated at 37 °C for 18 h. Inhibition of bacterial growth was measured in mm. Tests were performed in triplicate. Commercial discs of Ceftazidime (CAZ, 30 μg) were used as a reference.

In addition, a microdilution test was performed to determine the Minimum Inhibitory Concentration (MIC) and the Minimum Bactericidal Concentration (MBC). Serial dilutions of the EOs dissolved in DMSO were performed to obtain different concentration rates (from 40 to 0.07%(*v*/*v*)). Then, 50 μL of each dilution added to 50 μL of MHB was inoculated with 50 μL of bacteria broth adjusted to 0.5 McFarland (10^6^ CFU/mL) using 96-well microplates. After 24 h of incubation at 37 °C, MIC analysis was performed. In addition, 10 μL of each sample was subcultured onto MHA and incubated for 24 h at 37 °C to determine MBC. The concentration without visible growth was defined as the MIC, while the concentration without colony growth was considered as the MBC. Additionally, analysis for the MIC and MBC of antibiotics were performed using a concentration range from 256 to 0.25 μg/mL for CAZ and GEN and from 32 to 0.03 for CIP. It is important to remark that DMSO does not inhibit bacterial growth (data not shown).

### 4.5. Synergy between EOs and Antibiotics

For synergy analysis, a checkerboard assay was performed MHB with each EO. Oils were used at subinhibitory concentration dilutions (one quarter of the MIC was used), while antibiotics were used at the gradients described in the Materials and Methods section. Then, the assay was performed as previously described [51]. The fractional inhibitory concentration (FIC) for each EO and antibiotic was calculated by dividing the MIC of two drugs in combination with the MIC of each drug alone. The FIC Index (FICI), the sum of the FICs of each drug, was used to confirm the interaction of the two drugs. The FICI was considered as a synergistic effect if its value was ≤0.5, additive if it was from >0.5 to ≤1, indifferent if it was from >1.0 to ≤4, and antagonistic if it was >4. The experiments were carried out in triplicate, and results were expressed as the arithmetic mean of the three determinations.

### 4.6. EO Activity on Hypermucoviscosity Phenotype of the Strains

To identify the effect of EOs on hypermucoviscosity of the strains, they were analyzed by string test. Fresh colonies were cultured overnight on blood agar plates in subinhibitory concentrations of each EO and without them. Then, colonies were stretched outward by gently touching them with a loop, and the mucus filament length was measured.

### 4.7. Statistical Analysis

Results of this study are presented using means and standard deviations of triplicate assays. ANOVA and Tukey’s HSD post hoc analysis of variance were used to manage multiple comparisons. A significant difference was defined when *p* value was less than 0.05.

## 5. Conclusions

**Figure 7:**
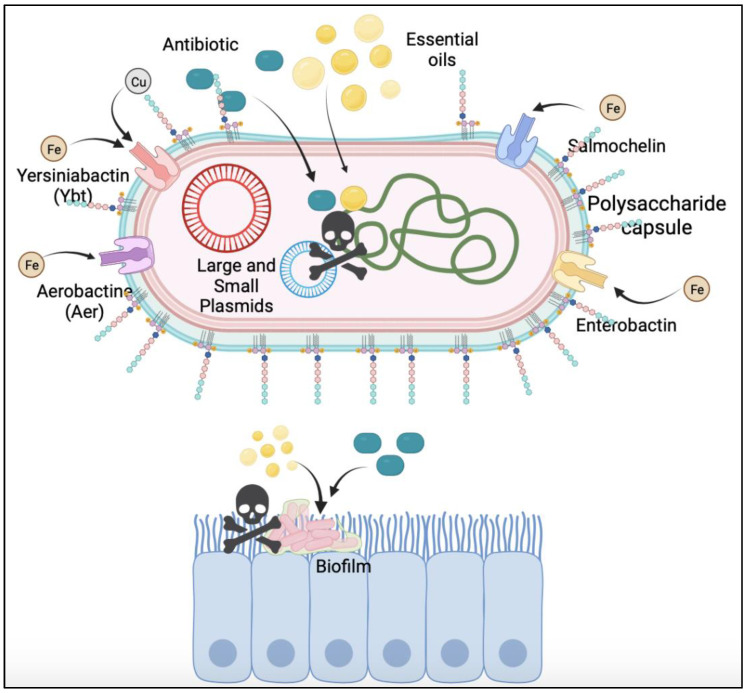
Mechanism of Action of the Synergic Effect of EOs with Antibiotics. The liposoluble nature of EOs affects the components involved with hypermucoviscosity phenotype (biofilm, capsule, siderophores, Lipopolysaccharides), which act as a physical barrier for antibiotics. When the hypermucoviscosity phenotype is affected, antibiotics could enter in the bacteria, and in synergy with EOs, induce bacteria death.

Antibiotics are considered the best therapy to combat bacterial infections, such as those caused by Kp. However, the rapid increase in resistance requires the development of new alternative therapies or synergistic therapies that help us combat these diseases. Essential oils represent a great tool in the development of synergistic therapies. This study demonstrates the potential of thyme, rosemary, and mint essential oils as synergistic therapies against Kp. Essential oils alter the hypermucoviscosity phenotype of the strains, breaking the lipid-soluble barrier that prevents the internalization and action of antibiotics (Figure 7). Once these factors are modified, antibiotics and essential oils can enter the pathogen and induce its death. This finding will allow us to develop a synergistic therapy to combat the infection caused by Kp regardless of the level of virulence, its resistance capacity, or hypermucoviscosity phenotype.

## Figures and Tables

**Table 1:** Essential oils included in this study.

Common Name	Scientific Name	Part Used	Extraction Method
Thyme	*Thymus vulgaris*	Leaf	Steam Distillation
Peppermint	*Mentha piperita*	Leaf/stem	Steam Distillation
Rosemary	*Rosmarinus officinalis*	Flower, leaf	Steam Distillation
Turmeric	*Curcuma longa*	Rhizome	Steam distillation

**Table 2:** Major constituents of thyme, rosemary, turmeric, and peppermint EOs identified by GC/MS analysis.

Essential Oil	Compound *	Retention Time	Match	% Area	Standard	IK exp	IK rep
		(min)					
**Thyme**	Thymol	13.33	98.4	37.31	Yes	1296	1291
	o-Cymene	8.4	98.9	22.12	No	1029	1022
	γ-Terpinene	9.06	99.5	11.97	Yes	1063	1060
	Caryophyllene	15.59	98.8	4.37	No	1433	1419
	Linalool	9.81	98.4	2.75	Yes	1102	1099
	Carvacrol	13.47	97.4	2.43	Yes	1304	1299
	β-Myrcene	7.7	98.3	1.78	No	993	991
	(+)-4-Carene	8.23	96.9	1.66	No	1020	1009
	Methyl carvacrol	12.5	95.9	1.25	No	1249	1244
	1-Octen-3-ol	7.45	96.9	1.22	No	980	980
	α-Pinene	6.61	96.5	1.16	Yes	937	937
	Camphene	6.9	98.1	1.12	No	952	951
	Borneol	11.33	98.1	1.06	No	1183	1167
	Camphor	10.74	97.5	0.91	No	1151	1142
	D-Limonene	8.47	91.6	0.71	Yes	1032	1018
	trans-4-Thuja-nol	9.22	94.5	0.71	No	1071	1070
	Caryophyllene oxide	18.13	88.2	0.64	No	1600	1581
	α-Thujene	6.47	94.8	0.49	No	930	929
	(+)-δ-Cadinene	17.16	82.4	0.35	No	1534	1524
	Eucalyptol	8.53	86.4	0.3	Yes	1036	1032
	(+)-epi-Bicyclosesquiphellandrene	17.05	84.3	0.26	No	1527	1435
	p-Mentha-2,4-(8)-diene	9.63	88.2	0.25	No	1092	1004
**Rosemary**	Eucalyptol	8.54	99.8	43.28	Yes	1036	1032
	(+)-2-Bornano-ne	10.74	99.4	13.34	No	1151	1143
	α-Pinene	6.61	99.3	13.17	Yes	937	937
	Caryophyllene	15.59	99.3	4.97	No	1433	1419
	β-Pinene	7.46	97.5	4.67	Yes	981	943
	Camphene	6.9	98.7	4.54	No	952	951
	Borneol	11.33	97.2	3.51	No	1172	1167
	D-Limonene	8.48	86	2.16	Yes	1033	1018
	α-Terpineol	11.56	90.04	2.15	Yes	1195	1189
	o-Cymene	8.39	97.7	1.53	No	1028	1022
	β-Myrcene	7.7	98.2	1.16	No	993	991
	Linalool	9.81	95.6	0.97	Yes	1102	1099
	Bornyl acetate	13.27	95.8	0.71	No	1293	1284
	Terpinen-4-ol	11.33	90.04	0.69	Yes	1183	1182
	δ-Terpinene	9.06	96.1	0.67	Yes	1063	1060
	(+)-4-Carene	8.23	96.4	0.52	No	1020	1009
	Humulene	16.13	86.2	0.41	No	1467	1454
	Isoterpinolene	9.64	91.7	0.31	No	1093	1004
	β-Phellandre-ne	6.38	87.8	0.16	No	925	1031
	α-Thujene	6.47	90.01	0.15	No	930	929
**Peppermint**	Menthol	11.26	99.8	38.19	No	1179	1164
	D-menthone	10.91	99.3	22.96	No	1160	1166
	p-Menthan-1-ol	11.09	95.2	8.39	No	1170	1178
	Eucalyptol	8.53	99.2	6.09	Yes	1036	1032
	D-Limonene	8.47	96.8	2.15	Yes	1032	1018
	Caryophyllene	15.59	97.3	1.81	No	1433	1419
	β-Myrcene	7.7	82.5	1.78	No	993	991
	Pulegone	12.47	97.8	1.67	No	1247	1237
	L-terpinen-4-ol	11.34	93	1.3	No	1183	1182
	β-Pinene	7.46	93.7	1.23	Yes	981	943
	α-Pinene	6.61	95.5	0.83	Yes	937	937
	dl-menthol	11.44	94.7	0.81	No	1189	1174
	Caryophyllene oxide	18.13	87.4	0.68	No	1600	1581
	o-Cymene	8.38	95.3	0.67	No	1028	1022
	Piperitone	12.72	94.3	0.59	No	1261	1253
	α-Terpineol	11.57	87.7	0.56	Yes	1196	1189
	Mintlactone	16.77	80.8	0.52	No	1508	1500
	δ-Terpinene	9.06	92.4	0.47	Yes	1063	1060
	Cadina-1(6),4-diene	17.16	86.9	0.4	No	1534	1469
	Linalool	9.81	83.6	0.31	Yes	1102	1099
	β-Bourbonene	15.01	89.6	0.27	No	1396	1384
	α-Thujene	6.47	92.1	0.25	No	930	929
	3-Octanol	7.78	88.9	0.25	No	997	994
	cis-Muurola-4(14),5-diene	16.45	83.5	0.25	No	1487	1435
**Turmeric**	ar-Turmerone	19.22	97.7	40.4	No	1677	1644
	Tumerone	19.28	98	16.36	No	1682	1632
	α-Curcumene	16.5	99	4.53	No	1491	1483
	Zingiberene	16.69	97.3	4.25	No	1503	1495
	β-Sesquiphellandrene	17.14	97.1	3.15	No	1533	1524
	(E)-Atlantone	20.7	97.6	2.54	No	1786	1773
	β-Bisabolene	16.89	96.9	1.25	No	1516	1509
	(Z)-γ-Atlanto-ne	19.59	91.5	0.85	No	1704	1699
	γ-Curcumene	16.45	85.7	0.75	No	1487	1480
	Caryophyllene	15.59	91.1	0.67	No	1433	1419
	7-epi-Bisabol-1-one	20.33	92	0.63	No	1759	1747
	o-Cymene	8.38	96.4	0.53	No	1028	1022
	Dicumene	16.84	83.6	0.48	No	1513	2021
	Eucalyptol	8.53	88.2	0.38	Yes	1036	1032
	2-Cyclohexen-1-one,3,4,4-tri-methyl	17.65	81.8	0.31	No	1568	1198
	α-Phellandre-ne	7.99	93.5	0.26	No	1008	1005
	Humulene	16.13	81.8	0.26	No	1467	1454
	(+)-4-Carene	9.64	88.6	0.21	No	1093	1009

* The essential oil constituents were characterized by matching their mass spectra with compound library NIST. IK exp—Kováts retention index experimental. IK rep—Kováts retention index reported.

**Table 3:** Fractional Inhibitory Concentration Index (FICI) of Ceftazidime and EOs against *Klebsiella pneumoniae* strains. The FICI was considered to indicate a synergistic effect if its value was ≤0.5, additive if it was from >0.5 to ≤1, indifferent if it was from >1.0 to ≤4, and antagonistic if it was >4.

Kp Strain	640U	889U	126U	98LCR	338U	537U1	971U	197U	182D	ATCC 700603	Mean FICI
CAZ + thyme	0.333	0.333	0.083	0.333	0.083	0.333	0.333	0.333	0.167	0.167	0.250
CAZ + turmeric	0.625	0.500	0.750	0.508	0.281	0.516	0.252	0.502	0.516	1.000	0.545
CAZ + rosemary	0.750	0.500	1.000	0.508	0.266	0.516	0.254	0.502	0.502	0.563	0.536
CAZ + peppermint	0.375	0.500	0.313	0.266	0.313	0.266	0.252	0.500	0.250	0.531	0.357

**Table 4:** Fractional Inhibitory Concentration Index (FICI) of Gentamicin (GEN) and EOs against *Klebsiella pneumoniae* strains. The FICI indicated a synergistic effect if its value was ≤0.5, additive if it was from >0.5 to ≤1, indifferent if it was from >1.0 to ≤4, and antagonism if it was >4.

Kp Strain	889U	98LCR	197U	Mean FICI
GEN + thyme	0.333	0.333	0.563	0.410
GEN + turmeric	2.250	0.375	0.625	1.083
GEN + rosemary	0.250	0.500	0.625	0.458
GEN + peppermint	0.060	0.250	0.516	0.275

**Table 5:** Fractional Inhibitory Concentration Index (FICI) of Ciprofloxacin (CIP) and EOs against *Klebsiella pneumoniae* strains. The FICI was the synergistic effect if its value was ≤0.5, additive if it was from >0.5 to ≤1, indifferent if it was from >1.0 to ≤4, and antagonism if it was >4.

Kp Strain	197U	817LCR	910LCR	Mean FICI
CIP + thyme	0.333	0.333	0.500	0.389
CIP + turmeric	0.750	1.250	0.500	0.835
CIP + rosemary	0.490	0.370	0.516	0.459
CIP + peppermint	0.180	0.250	0.50	0.311

**Table 6:** Strain resistance classification based on resistance patterns. LRS: Low Resistant Strain, MRS: Medium Resistant Strain, HRS: High Resistant Strain, CPD: cefpodoxime, CRO: ceftriaxone, CAZ: Ceftazidime, CEF: cefepime, CIP: ciprofloxacin, ATM: aztreonam, AMI: amikacin, GEN: Gentamycin, STX: trimethoprim/sulfamethoxazole. Breakpoints were established as reported by CLSI.

Strain	Resistance Classification	Resistant	Intermediate	Susceptible
640U	LRS	ATM	-	CPD, CRO, CAZ, CEF, CIP, AMI, GEN, STX
889U	LRS	STX	-	CPD, CRO, CAZ, CEF, CIP, ATM, AMI, GEN
126U	LRS	ATM	-	CPD, CRO, CAZ, CEF, CIP, AMI, GEN, STX
98LCR	MRS	CPD	CAZ, CRO, GEN	CEF, CIP, ATM, AMI, STX
338U	MRS	CPD, GEN	CRO, CAZ, ATM, STX	CEF, CIP, AMI
537U1	MRS	CPD	CRO, CAZ, ATM, AMI,	CEF, CIP, GEN, STX
971U	HRS	CPD, CRO, CAZ, CEF, ATM, AMI, GEN,		CIP, STX
197U	HRS	CPD, CRO, CAZ, CEF, ATM, AMI, GEN, STX		CIP
182D	HRS	CPD, CRO, CAZ, CEF, ATM, AMI,	STX	CIP, GEN

## Data Availability

Data is contained within the article.
